# Supplementary material for: Pyruvate Dehydrogenase Kinase 1 inhibition mediated oxidative phosphorylation enhancement in cartilage promotes osteoarthritis progression
Source: BMC Musculoskelet Disord. 2023 Jul 20;24:597. doi: 10.1186/s12891-023-06585-6 (PMC10357736; doi:10.1186/s12891-023-06585-6)
Supplement: Supplementary file 1 — Additional file 1. [file 12891_2023_6585_MOESM1_ESM.zip › KEGG.Gsea.1653622432464/KEGG_GRAFT_VERSUS_HOST_DISEASE.html]

Details for gene set KEGG\_GRAFT\_VERSUS\_HOST\_DISEASE[GSEA]

|  || Dataset | 55457\_collapsed\_to\_symbols.cls.cls #OA\_versus\_normal.cls.cls #OA\_versus\_normal\_repos |
| Phenotype | cls.cls#OA\_versus\_normal\_repos |
| Upregulated in class | normal |
| GeneSet | KEGG\_GRAFT\_VERSUS\_HOST\_DISEASE |
| Enrichment Score (ES) | -0.36425358 |
| Normalized Enrichment Score (NES) | -1.304322 |
| Nominal p-value | 0.125 |
| FDR q-value | 0.28016597 |
| FWER p-Value | 0.999 |
Table: GSEA Results Summary

  

Fig 1: Enrichment plot: KEGG\_GRAFT\_VERSUS\_HOST\_DISEASE      
 Profile of the Running ES Score & Positions of GeneSet Members on the Rank Ordered List

  

| SYMBOL | TITLE | RANK IN GENE LIST | RANK METRIC SCORE | RUNNING ES | CORE ENRICHMENT || 1 | HLA-DMB | "major histocompatibility complex, class II, DM beta [Source:HGNC Symbol;Acc:HGNC:4935]" | 679 | 0.598 | 0.0050 | No |
| 2 | CD86 | CD86 molecule [Source:HGNC Symbol;Acc:HGNC:1705] | 689 | 0.596 | 0.0618 | No |
| 3 | HLA-DQA1 | "major histocompatibility complex, class II, DQ alpha 1 [Source:HGNC Symbol;Acc:HGNC:4942]" | 1334 | 0.452 | 0.0554 | No |
| 4 | HLA-DMA | "major histocompatibility complex, class II, DM alpha [Source:HGNC Symbol;Acc:HGNC:4934]" | 1769 | 0.388 | 0.0591 | No |
| 5 | TNF | tumor necrosis factor [Source:HGNC Symbol;Acc:HGNC:11892] | 2098 | 0.350 | 0.0674 | No |
| 6 | HLA-DOB | "major histocompatibility complex, class II, DO beta [Source:HGNC Symbol;Acc:HGNC:4937]" | 2147 | 0.344 | 0.0969 | No |
| 7 | HLA-DQB1 | "major histocompatibility complex, class II, DQ beta 1 [Source:HGNC Symbol;Acc:HGNC:4944]" | 2840 | 0.266 | 0.0688 | No |
| 8 | FAS | Fas cell surface death receptor [Source:HGNC Symbol;Acc:HGNC:11920] | 3828 | 0.173 | 0.0089 | No |
| 9 | FASLG | Fas ligand [Source:HGNC Symbol;Acc:HGNC:11936] | 3931 | 0.162 | 0.0166 | No |
| 10 | HLA-DOA | "major histocompatibility complex, class II, DO alpha [Source:HGNC Symbol;Acc:HGNC:4936]" | 4344 | 0.125 | -0.0034 | No |
| 11 | CD28 | CD28 molecule [Source:HGNC Symbol;Acc:HGNC:1653] | 4543 | 0.110 | -0.0083 | No |
| 12 | KLRD1 | killer cell lectin like receptor D1 [Source:HGNC Symbol;Acc:HGNC:6378] | 4778 | 0.089 | -0.0179 | No |
| 13 | HLA-DPB1 | "major histocompatibility complex, class II, DP beta 1 [Source:HGNC Symbol;Acc:HGNC:4940]" | 5486 | 0.033 | -0.0696 | No |
| 14 | IL2 | interleukin 2 [Source:HGNC Symbol;Acc:HGNC:6001] | 5819 | 0.007 | -0.0947 | No |
| 15 | KIR3DL2 | "killer cell immunoglobulin like receptor, three Ig domains and long cytoplasmic tail 2 [Source:HGNC Symbol;Acc:HGNC:6339]" | 5893 | 0.002 | -0.1002 | No |
| 16 | GZMB | granzyme B [Source:HGNC Symbol;Acc:HGNC:4709] | 6132 | -0.015 | -0.1172 | No |
| 17 | IFNG | interferon gamma [Source:HGNC Symbol;Acc:HGNC:5438] | 6410 | -0.036 | -0.1353 | No |
| 18 | HLA-DRA | "major histocompatibility complex, class II, DR alpha [Source:HGNC Symbol;Acc:HGNC:4947]" | 6550 | -0.046 | -0.1417 | No |
| 19 | KLRC1 | killer cell lectin like receptor C1 [Source:HGNC Symbol;Acc:HGNC:6374] | 6835 | -0.066 | -0.1574 | No |
| 20 | CD80 | CD80 molecule [Source:HGNC Symbol;Acc:HGNC:1700] | 6918 | -0.074 | -0.1567 | No |
| 21 | HLA-DRB1 | "major histocompatibility complex, class II, DR beta 1 [Source:HGNC Symbol;Acc:HGNC:4948]" | 7087 | -0.086 | -0.1614 | No |
| 22 | HLA-F | "major histocompatibility complex, class I, F [Source:HGNC Symbol;Acc:HGNC:4963]" | 7196 | -0.096 | -0.1606 | No |
| 23 | IL1A | interleukin 1 alpha [Source:HGNC Symbol;Acc:HGNC:5991] | 7842 | -0.144 | -0.1968 | No |
| 24 | PRF1 | perforin 1 [Source:HGNC Symbol;Acc:HGNC:9360] | 8265 | -0.176 | -0.2126 | No |
| 25 | KIR2DL1 | "killer cell immunoglobulin like receptor, two Ig domains and long cytoplasmic tail 1 [Source:HGNC Symbol;Acc:HGNC:6329]" | 8348 | -0.182 | -0.2014 | No |
| 26 | HLA-DPA1 | "major histocompatibility complex, class II, DP alpha 1 [Source:HGNC Symbol;Acc:HGNC:4938]" | 9481 | -0.273 | -0.2631 | No |
| 27 | KIR2DL5A | "killer cell immunoglobulin like receptor, two Ig domains and long cytoplasmic tail 5A [Source:HGNC Symbol;Acc:HGNC:16345]" | 9695 | -0.291 | -0.2516 | No |
| 28 | HLA-G | "major histocompatibility complex, class I, G [Source:HGNC Symbol;Acc:HGNC:4964]" | 10869 | -0.413 | -0.3029 | No |
| 29 | HLA-E | "major histocompatibility complex, class I, E [Source:HGNC Symbol;Acc:HGNC:4962]" | 11659 | -0.526 | -0.3135 | Yes |
| 30 | KIR3DL1 | "killer cell immunoglobulin like receptor, three Ig domains and long cytoplasmic tail 1 [Source:HGNC Symbol;Acc:HGNC:6338]" | 11864 | -0.563 | -0.2750 | Yes |
| 31 | IL1B | interleukin 1 beta [Source:HGNC Symbol;Acc:HGNC:5992] | 12105 | -0.622 | -0.2336 | Yes |
| 32 | IL6 | interleukin 6 [Source:HGNC Symbol;Acc:HGNC:6018] | 12460 | -0.730 | -0.1907 | Yes |
| 33 | HLA-A | "major histocompatibility complex, class I, A [Source:HGNC Symbol;Acc:HGNC:4931]" | 12502 | -0.749 | -0.1216 | Yes |
| 34 | HLA-B | "major histocompatibility complex, class I, B [Source:HGNC Symbol;Acc:HGNC:4932]" | 12534 | -0.764 | -0.0503 | Yes |
| 35 | HLA-DRB4 | "major histocompatibility complex, class II, DR beta 4 [Source:HGNC Symbol;Acc:HGNC:4952]" | 12636 | -0.815 | 0.0205 | Yes |
Table: GSEA details [plain text format]

  

Fig 2: KEGG\_GRAFT\_VERSUS\_HOST\_DISEASE      
 Blue-Pink O' Gram in the Space of the Analyzed GeneSet

  

Fig 3: KEGG\_GRAFT\_VERSUS\_HOST\_DISEASE: Random ES distribution      
 Gene set null distribution of ES for **KEGG\_GRAFT\_VERSUS\_HOST\_DISEASE**
